# Adverse event profiles of dipeptidyl peptidase-4 inhibitors: data mining of the public version of the FDA adverse event reporting system

**DOI:** 10.1186/s40360-020-00447-w

**Published:** 2020-09-16

**Authors:** Jing Huang, Yuntao Jia, Shusen Sun, Long Meng

**Affiliations:** 1 Department of Respiratory and Critical Care Medicine, The First Affiliated Hospital of Chongqing Medical University, Chongqing, 400016 China; 2 Department of Pharmacy, Ministry of Education Key Laboratory of Child Development and Disorders, China International Science and Technology Cooperation Base of Child Development and Critical Disorders, Chongqing Key Laboratory of Pediatrics, Children’s Hospital of Chongqing Medical University, Chongqing, China; 3 Department of Pharmacy Practice, College of Pharmacy and Health Sciences, Western New England University, 1215 Wilbraham Road, Springfield, USA; 4 Department of Pharmacy, Xiangya Hospital Central South University, Changsha, Hunan China; 5 Institute for Rational and Safe Medication Practices, National Clinical Research Center for Geriatric Disorders, Xiangya Hospital, Central South University, Changsha, Hunan China; 6 Department of Pharmacy, The First Affiliated Hospital of Chongqing Medical University, Chongqing, China

**Keywords:** Pharmacovigilance, Data mining, Dipeptidyl Peptidase-4 inhibitors, Adverse event reporting system

## Abstract

**Background:**

To describe and analyze the patterns of adverse events associated with dipeptidyl peptidase-4 inhibitors (DPP-4is) (sitagliptin, saxagliptin, linagliptin, vildagliptin, and alogliptin) from the FDA Adverse Event Reporting System (FAERS) and to highlight areas of safety concerns.

**Methods:**

Adverse events spontaneously submitted to the FAERS between 2004 Q1 to 2019 Q2 were included. The online tool OpenVigil 2.1 was used to query the database. The research relied on definitions of preferred terms (PTs) specified by the Medical Dictionary for Regulatory Activities (MedDRA) and the standardized MedDRA Queries (SMQ). The reporting odds ratio (ROR), with 95% confidence intervals (CIs) was calculated for disproportionality analysis.

**Results:**

Over 16 years, a total of 9706 adverse event reports were identified. Alogliptin was excluded from further analysis due to insufficient sample size. Compared with the non-insulin antidiabetic drugs, the four DPP-4is were all disproportionately associated with four SMQs: “gastrointestinal nonspecific inflammation and dysfunctional conditions,” “hypersensitivity,” “severe cutaneous adverse reactions,” and “noninfectious diarrhoea”. As for PT level analyses, DPP-4is are associated with higher reporting of the gastrointestinal tract, pancreas, malignancies, infection, musculoskeletal disorders, general disorders, hypersensitivity, and skin AEs.

**Conclusions:**

Data mining of the FAERS is useful for examining DPP-4 inhibitors-associated adverse events. The findings of the present study are compatible with clinical experience, and it provides valuable information to decision-makers and healthcare providers in clinical practice.

## Background

Type 2 diabetes mellitus (T2DM) is the most common form of diabetes. Oral agents are the mainstay of pharmacological treatment for T2DM. Dipeptidyl peptidase-4 inhibitors (DPP-4is) are a valuable addition to the antidiabetic treatment modalities and have been widely used [1]. DPP-4is, sitagliptin, saxagliptin, linagliptin, vildagliptin, and alogliptin, have significantly different chemical structures, leading to differences in their pharmacokinetic and pharmacodynamic properties. It is not entirely clear if these differences may result in differing safety profiles [2]. The drugs’ safety profiles in clinical practice may differ from clinical trials that have been well-designed [3]. Therefore, it is necessary to explore adverse events (AEs) induced by DPP-4is in a real-world environment.

Spontaneous reporting systems (SRS) of AEs generate large pharmacovigilance databases, which can be used for safety assessments on drug utilization in clinical practice [4]. The FDA Adverse Event Reporting System (FAERS), an open information database, was established to serve the FDA’s post-marketing monitoring programs for drugs and therapeutic products. The AE reports are evaluated through quantitative signal detection algorithms, where a signal is an indicator of possible real safety issues [5]. Signal detection is one of the essential tools of pharmacovigilance [6].

Previous pharmacovigilance studies have revealed the association between DPP-4is and some particular adverse events [7–9]. To achieve the latest information about the safety profiles of DPP-4is, we queried an international SRS, namely FAERS, to characterize the reporting pattern of DPP-4is and map the entire spectrum of AEs by the pharmacovigilance approach.

## Methods

### Data sources

Five DPP-4is, sitagliptin, saxagliptin, linagliptin, vildagliptin, and alogliptin, were selected as study drugs. Data from the SRS database were fully anonymized by regulatory authorities.

Data for this study were retrieved from the public release of the FAERS database, which covered the period from January 1, 2004, to June 30, 2019. FAERS is updated quarterly by the FDA, and it is one of the most extensive public databases in the world. To mine the FAERS, we used OpenVigil FDA, a validated pharmacovigilance tool, to retrieve FAERS data through the openFDA application programming interface for evaluating the FDA drug-event database with the additional openFDA duplicate detection and drug mapping functionality [10, 11], and it is used in many pharmacovigilance studies [12, 13]. OpenVigil operates only on the cleaned FDA data by deleting duplicates or reports with missing data [11].

### Definition of adverse events

Adverse events in the FAERS database are coded according to the preferred terms (PTs) derived from the Medical Dictionary for Regulatory Activities (MedDRA) terminology. Besides, different PTs can also be combined to define a medical condition or area of interest through an algorithmic approach known as the Standardized MedDRA Queries (SMQs). The SMQs and PTs are extensively accepted and used in FAERS data analysis.

### Disproportionality analyses

As a fundamental analytic method of pharmacovigilance, disproportionality analysis, which compares the proportion of occurring AEs between a specific drug and all other drugs, was applied to identify drug-associated adverse events as signals [14].

Firstly, we conducted a disproportionality analysis using all existing narrow SMQs to map the safety profiles of DPP-4is. Key toxicities that emerged from events with significant disproportionality in the first step were characterized in terms of specific signs/symptoms (PT level). Further disproportionality analysis of PT levels was based on the signal strength, literature and number of reported AEs associated with DPP-4is in different systems to examine the safety profile of DPP-4is [12].

To mitigate the confounding effects of “indication bias” (i.e., the indication for which the drug is prescribed is reported as an AE), PTs and SMQs associated with diabetes-related signs and complications were removed from analysis.

In every FAERS AE report, role codes had been assigned by reporters to each reported drug and indicated as either primary suspect drug, secondary suspect drug, concomitant or interacting. In our study, a case/non-case method was conducted for thedisproportionality analyses. Cases defined as AEs reports in which the reporter mentioned DPP-4is as suspect (“Primary Suspect”, “Secondary Suspect” or “Concomitant”) were selected. To control for major reporting and confounders, non-cases were selected by restricting the analysis to AEs reports in which at least one antidiabetic agent (excluding insulins) was recorded (ATC code: A10B), the so-called analysis by therapeutic area [15, 16].

In this study, the signals of disproportionate reporting (SDR) were generated by calculating the reporting odds ratio (ROR). ROR is a pharmacovigilance index that is clear and easy to understand, and it is widely used in epidemiological studies. The value ROR was calculated using the equation (ROR = (a × d)/(b × c)) and expressed as point estimates and its two-sides 95% confidence interval (CI) (Supplementary Table 1). When a specific drug is more likely to induce a specific AE than all other drugs, it would typically receive a higher ROR value due to higher disproportionality. SDR is considered significant when the lower limit of the 95% CI of the ROR estimates is higher than one and at least three cases of interest reported [17]. The analyses were conducted using the Microsoft EXCEL 2010 and SPSS 23.0 statistical software.

## Results

During the study period, the FAERS database received 9706 DPP-4i-associated adverse event reports (AERs): 680 for saxagliptin, 7811 for sitagliptin, 802 for linagliptin, 347 for vildagliptin, and 66 for alogliptin. Because the total number of adverse events occurring with alogliptin was not large enough to compare the association with adverse events of the other four DPP-4is, alogliptin was excluded from further analysis [4]. Table 1 shows event numbers and patient demographic characteristics due to these adverse events.

**Table 1:** Characteristics of adverse event reports

	Saxagliptin (%)	Sitagliptin (%)	Linagliptin (%)	Vildagliptin (%)
**Number of events**	680	7811	802	347
**Gender**				
Female	313 (46)	3703 (47)	379 (47)	159 (46)
Male	340 (50)	3233 (41)	360 (45)	171 (49)
Unknown	27 (4)	875 (11)	63 (8)	17 (5)
**Age (year)**				
<18	0 (0)	8 (0)	3 (0)	0 (0)
18–44	35 (5)	39 (0)	31 (4)	18 (5)
45–64	273 (40)	2286 (29)	169 (21)	91 (26)
65–74	143 (21)	3386 (43)	166 (21)	70 (20)
≥ 75	98 (14)	1000 (13)	171 (21)	82 (24)
Unknown	131 (19)	1092 (14)	262 (33)	86 (25)
**Outcome of AEs**				
Hospitalization (initial or prolonged)	188 (28)	2198 (28)	253 (32)	149 (43)
Disability	18 (3)	373 (5)	30 (4)	11 (3)
Life-threatening	23 (3)	473 (6)	36 (4)	21 (6)
Death	25 (4)	483 (6)	37 (5)	50 (14)

Table 2 lists the SMQs most frequently reported with the use of saxagliptin, sitagliptin, linagliptin, and vildagliptin, which were ranked according to the number of reports. Considering DPP-4is as a class, SDR emerged in 13 SMQs: “gastrointestinal nonspecific inflammation and dysfunctional conditions”, “hypersensitivity”, “acute pancreatitis”, “haemodynamic oedema, effusions and fluid overload”, “malignancies”, “noninfectious diarrhoea”, “angioedema”, “hepatic disorders”, “arthritis”, “gastrointestinal perforation, ulceration, haemorrhage or obstruction”, “severe cutaneous adverse reactions”, “taste and smell disorders” and “anaphylactic reaction”. The SDR was most commonly observed in the following systems/organs: gastrointestinal, hepatic, musculoskeletal, malignancies, pancreas, hypersensitivity, and skin. The following five SMQs emerged statistically significantly in these four agents RORs in: “gastrointestinal nonspecific inflammation and dysfunctional conditions”, “hypersensitivity”, “severe cutaneous adverse reactions” and “noninfectious diarrhoea”.

**Table 2:** The top 20 most frequently reproted SMQs of DPP-4is

SMQs		Saxagliptin		Sitagliptin		Linagliptin		Vildagliptin		DPP-4is	
		N	ROR(95% CI)	N	ROR(95% CI)	N	ROR(95% CI)	N	ROR(95% CI)	N	ROR(95% CI)
Gastrointestinal nonspecific inflammation and dysfunctional conditions		114	4.27 (3.48,5.23)^*^	948	3.23 (3.00,3.48)^*^	86	2.53 (2.02,3.17)^*^	37	2.50 (1.78,3.52)^*^	1180	3.39 (3.17,3.63)^*^
Hypersensitivity		113	8.04 (6.55,9.87)^*^	732	4.91 (4.51,5.35)^*^	68	3.68 (2.86,4.73)^*^	22	2.66 (1.73,4.10)^*^	932	5.47 (5.05,5.91)^*^
Acute pancreatitis		79	11.27 (8.87,14.33)^*^	738	14.99 (13.54,16.58)^*^	42	4.62 (3.37,6.33)^*^	7	1.69 (0.80,3.58)	862	16.32 (14.76,18.04)^*^
Haemodynamic oedema, effusions and fluid overload		40	2.26 (1.64,3.11)^*^	329	1.64 (1.46,1.84)^*^	23	1.06 (0.70,1.61)	21	2.32 (1.49,3.61)^*^	411	1.68 (1.52,1.87)^*^
Malignancies		21	1.97 (1.27,3.05)^*^	346	3.22 (2.86,3.62)^*^	34	2.76 (1.95,3.90)^*^	9	1.64 (0.85,3.19)	405	3.12 (2.79,3.48)^*^
Noninfectious diarrhoea		44	4.28 (3.15,5.83)^*^	250	2.15 (1.88,2.46)^*^	28	2.22 (1.52,3.25)^*^	20	3.75 (2.38,5.91)^*^	341	2.48 (2.20,2.79)^*^
Angioedema		52	10.82 (8.10,14.46)^*^	254	5.21 (4.52,6.01)^*^	30	4.96 (3.43,7.19)^*^	3	1.09 (0.35,3.40)	337	6.20 (5.44,7.07)^*^
Embolic and thrombotic events		10	0.05 (0.03,0.09)	180	0.07 (0.06,0.09)	30	0.13 (0.09,0.19)	19	0.20 (0.12,0.31)	239	0.08 (0.07,0.09)
Hepatic disorders		15	3.53 (2.11,5.92)^*^	148	3.40 (2.85,4.07)^*^	8	1.56 (0.78,3.15)	10	4.63 (2.46,8.72)^*^	179	3.45 (2.92,4.08)^*^
Cardiac failure		11	0.11 (0.06,0.21)	122	0.10 (0.09,0.12)	23	0.20 (0.13,0.31)	12	0.25 (0.14,0.44)	167	0.11 (0.10,0.13)
Hypertension		15	1.21 (0.73,2.02)	118	0.81 (0.68,0.98)	6	0.40 (0.18,0.90)	27	4.57 (3.08,6.78)^*^	166	0.94 (0.80,1.10)
Chronic kidney disease		7	0.67 (0.32,1.41)	137	1.16 (0.98,1.39)	12	0.98 (0.55,1.74)	7	1.33 (0.63,2.82)	162	1.12 (0.95,1.31)
Arthritis		6	1.93 (0.86,4.34)	79	2.39 (1.88,3.03)^*^	10	2.76 (1.47,5.17)^*^	3	1.89 (0.60,5.90)	97	2.43 (1.96,3.03)^*^
Gastrointestinal perforation, ulceration, haemorrhage or obstruction		6	1.60 (0.71,3.59)	66	1.58 (1.23,2.04)^*^	11	2.52 (1.38,4.58)^*^	7	3.72 (1.75,7.89)^*^	90	1.79 (1.44,2.24)^*^
Dyslipidaemia		8	1.38 (0.68,2.78)	70	1.05 (0.82,1.34)	2	0.29 (0.07,1.15)	3	1.01 (0.32,3.15)	82	1.00 (0.79,1.25)
Severe cutaneous adverse reactions		6	4.84 (2.14,10.91)^*^	62	5.34 (4.01,7.13)^*^	8	5.51 (2.72,11.19)^*^	4	6.30 (2.33,17.01)^*^	80	6.12 (4.69,8.00)^*^
Depression and suicide/self-injury		9	1.74 (0.90,3.37)	60	1.00 (0.77,1.30)	6	0.97 (0.44,2.18)	5	1.89 (0.78,4.59)	80	1.09 (0.87,1.37)
Retinal disorders		4	1.29 (0.48,3.45)	34	0.95 (0.67,1.34)	7	1.92 (0.91,4.07)	3	1.90 (0.61,5.93)	46	1.05 (0.78,1.42)
Taste and smell disorders		0	–	37	2.82 (1.99,4.00)^*^	2	1.34 (0.33,5.40)	4	6.32 (2.34,17.08)^*^	43	2.70 (1.94,3.76)^*^
Anaphylactic reaction		11	7.23 (3.94,13.26)^*^	25	1.40 (0.93,2.11)	3	1.61 (0.51,5.02)	3	3.74 (1.20,11.73)^*^	39	2.01 (1.45,2.78)^*^

*Dpp-4is* Dipeptidyl peptidase-4 inhibitors

*N* The number of adverse events reports

*ROR* The reporting odds ratio

*CI* The confidence interval

*SMQs* Standardized MedDRA queries

^*^: signal detected, see “Methods” for the criteria of detection

Then the combined analysis of FAERS data with literature reviews identified 30 PTs (involved in eight systems/organs), which were further explored. We found statistically-significant RORs for eight organs/systems of AEs, including gastrointestinal, pancreatitis, malignancies, infection, musculoskeletal, general disorders, hypersensitivity, and skin AEs. Furthermore, a signal was not detected for cardiopathy (Table 3).

**Table 3:** Signal strength for DPP-4is at the PT level in FAERS

	PTs	Saxagliptin		Sitagliptin		Linagliptin		Vildagliptin		DPP-4is	
		N	ROR(95% CI)	N	ROR(95% CI)	N	ROR(95% CI)	N	ROR(95% CI)	N	ROR(95% CI)
Gastrointestinal	Diarrhoea	42	4.17 (3.04,5.71)^*^	245	2.15 (1.88,2.46)^*^	23	1.85 (1.22,2.81)^*^	20	3.84 (2.44,6.04)^*^	329	2.44 (2.16,2.75)^*^
	Nausea	40	3.70 (2.68,5.11)^*^	327	2.82 (2.50,3.18)^*^	35	2.69 (1.91,3.79)^*^	14	2.47 (1.44,4.22)^*^	413	3.00 (2.69,3.34)^*^
	Vomiting	15	2.02 (1.21,3.38)^*^	160	1.97 (1.67,2.32)^*^	12	1.36 (0.77,2.40)	14	3.77 (2.20,6.46)^*^	200	2.04 (1.75,2.37)^*^
	Dyspepsia	10	5.54 (2.94,10.44)^*^	50	2.55 (1.89,3.44)^*^	7	3.24 (1.53,6.87)^*^	1	1.05 (0.15,7.49)	68	2.95 (2.27,3.85)^*^
Pancreas	Acute pancreatitis	26	11.80 (7.89,17.65)^*^	119	5.50 (4.46,6.78)^*^	8	2.87 (1.42,5.80)^*^	6	5.01 (2.22,11.29)^*^	159	6.66 (5.50,8.07)^*^
Malignancies	Pancreatic carcinoma	1	0.92 (0.13,6.58)	122	22.04 (16.77,28.97)^*^	4	3.18 (1.18,8.57)^*^	1	1.81 (0.25,12.97)	127	19.44 (14.76,25.60)^*^
Infection	Nasopharyngitis	8	5.97 (2.94,12.12)^*^	34	2.30 (1.61,3.30)^*^	4	2.48 (0.92,6.66)	1	1.42 (0.20,10.12)	47	2.70 (1.97,3.71)^*^
	Pneumonia	6	1.47 (0.65,3.29)	85	1.90 (1.52,2.39)^*^	11	2.30 (1.26,4.19)^*^	10	4.92 (2.62,9.27)^*^	112	2.09 (1.71,2.56)^*^
	Upper respiratory tract infection	6	8.52 (3.75,19.36)^*^	71	15.71 (11.32,21.80)^*^	1	1.15 (0.16,8.25)	0	–	78	15.30 (11.01,21.24)^*^
	Urinary tract infection	5	1.79 (0.74,4.33)	62	2.04 (1.57,2.66)^*^	7	2.13 (1.01,4.51)^*^	6	4.27 (1.90,9.61)^*^	79	2.16 (1.70,2.75)^*^
Musculoskeletal	Arthralgia	13	3.46 (1.99,6.02)^*^	145	3.87 (3.22,4.64)^*^	10	2.23 (1.19,4.18)^*^	7	3.63 (1.71,7.71)^*^	173	3.88 (3.27,4.60)^*^
	Pain in extremity	10	1.96 (1.05,3.68)^*^	113	2.04 (1.67,2.48)^*^	10	1.66 (0.89,3.11)	6	2.31 (1.03,5.20)^*^	138	2.05 (1.72,2.46)^*^
	Myalgia	9	3.00 (1.55,5.82)^*^	133	4.64 (3.82,5.64)^*^	15	4.30 (2.56,7.21)^*^	3	1.93 (0.62,6.04)	159	4.71 (3.93,5.66)^*^
	Fracture	0	–	9	1.22 (0.62,2.41)	0	–	1	3.04 (0.42,21.80)	10	1.10 (0.58,2.09)
Cardiac	Cardiac failure	5	1.00 (0.42,2.43)	43	0.74 (0.54,1.00)	9	1.54 (0.80,2.99)	5	1.99 (0.82,4.82)	61	0.86 (0.66,1.11)
	Cardiac failure congestive	5	0.06 (0.02,0.14)	53	0.05 (0.04,0.06)	6	0.06 (0.03,0.13)	1	0.02 (0,0.16)	65	0.05 (0.04,0.06)
	Myocardial infarction	3	0.02 (0.01,0.06)	122	0.06 (0.05,0.08)	14	0.08 (0.04,0.13)	10	0.13 (0.07,0.24)	149	0.06 (0.05,0.07)
Hypersensitivity and skin	Angioedema	20	27.80 (17.30,44.65)^*^	50	7.06 (5.06,9.86)^*^	5	5.21 (2.13,12.72)^*^	0	–	75	10.90 (8.01,14.83)^*^
	Oedema peripheral	19	3.95 (2.49,6.26)^*^	124	2.36 (1.95,2.85)^*^	1	0.17 (0.02,1.20)	13	5.32 (3.05,9.30)^*^	156	2.47 (2.08,2.94)^*^
	Pruritus	13	3.94 (2.26,6.86)^*^	138	4.26 (3.53,5.14)^*^	17	4.40 (2.70,7.16)^*^	4	2.33 (0.87,6.26)	171	4.52 (3.79,5.38)^*^
	Rash	38	9.76 (6.99,13.64)^*^	240	6.63 (5.70,7.72)^*^	10	2.01 (1.07,3.76)^*^	6	2.80 (1.24,6.29)^*^	294	7.17 (6.21,8.28)^*^
	Rash generalised	3	4.43 (1.41,13.96)^*^	42	7.27 (5.04,10.47)^*^	7	9.08 (4.23,19.49)^*^	0	–	52	8.09 (5.71,11.44)^*^
	Urticaria	21	12.47 (7.97,19.51)^*^	74	4.20 (3.25,5.43)^*^	10	4.78 (2.54,6.90)^*^	0	–	104	5.31 (4.22,6.67)^*^
	Pemphigoid	3	8.37 (2.63,26.64)^*^	9	2.25 (1.12,4.53)^*^	20	63.72 (37.92,107.09)^*^	16	112.37 (63.87,197.70)^*^	47	23.33 (14.44,37.68)^*^
General disorders	Asthenia	8	1.36 (0.67,2.73)	111	1.71 (1.40,2.08)^*^	26	3.87 (2.61,5.75)^*^	4	1.33 (0.49,3.56)	148	1.89 (1.59,2.25)^*^
	Dizziness	15	1.75 (1.05,2.93)^*^	224	2.48 (2.15,2.86)^*^	25	2.51 (1.68,3.75)^*^	5	1.13 (0.47,2.74)	269	2.46 (2.16,2.81)^*^
	Fatigue	14	1.92 (1.13,3.27)^*^	141	1.74 (1.46,2.08)^*^	15	1.74 (1.04,2.91)^*^	5	1.33 (0.55,3.22)	175	1.78 (1.52,2.09)^*^
	Headache	19	2.87 (1.81,4.54)^*^	286	4.51 (3.95,5.15)^*^	22	2.82 (1.84,4.32)^*^	2	0.57 (0.14,2.30)	329	4.33 (3.82,4.91)^*^
	Malaise	14	2.26 (1.33,3.86)^*^	130	1.91 (1.59,2.29)^*^	13	1.77 (1.02,3.07)^*^	23	7.73 (5.04,11.84)^*^	180	2.22 (1.90,2.61)^*^
	Pain	18	2.18 (1.36,3.49)^*^	122	1.29 (1.07,1.55)^*^	13	1.32 (0.76,2.28)	1	0.23 (0.03,1.64)	154	1.33 (1.13,1.57)^*^

*FAERS* FDA Adverse Event Reporting System

*Dpp-4is* Dipeptidyl peptidase-4 inhibitors

*N* The number of adverse events reports

*ROR* The reporting odds ratio

*CI* The confidence interval

*PTs* Preferred Terms

^*^: signal detected, see “Methods” for the criteria of detection

## Discussion

In this study, AERs submitted to the FAERS database were reviewed to determine the safety profiles of DPP-4is. A lot of clinical trials were conducted to evaluate the safety of DPP-4i, and the results varied with study parameters. Overall, the results obtained herein were consistent with clinical trials, suggesting the usefulness of the FAERS database and the data mining methods.

Tissues that strongly express DPP-4 include the heart and blood vessels, muscle, exocrine pancreas, kidney, and lymph nodes. The ability of DPP-4i to inhibit enzymes in the DPP- family [18] or an off-target effect of the DPP-4i might be responsible for adverse drug reactions.

Gastrointestinal intolerance presenting as nausea, vomiting, diarrhoea, and dyspepsia [19–22] is one of the most commonly reported adverse reactions for DPP-4 [23]. According to a published study, the incidence of gastrointestinal adverse reactions was up to 12.2% [24]. The mechanism of DPP-4i associated gastrointestinal intolerance appears to be partially dependent on the motility effects of glucagon-like peptide-1 (GLP-1) elevated by DPP-4 inhibition and other gastric hormones, such as pituitary adenylate cyclase-activating peptide, gastric inhibitory polypeptide, and oxyntomodulin [25]. These drug-associated adverse event signals were detected for the four DPP-4is.

Significant signals of acute pancreatitis were detected in the four DPP-4is; significant signals of pancreatic carcinoma were also detected in DPP-4i(as a class) and sitagliptin, linagliptin (as a single drug). Consistent with our study, several animal studies, cases, and clinical data, suggested an increased risk of pancreatitis with the use of these drugs [26, 27]. Another pharmacovigilance study found DPP-4i is associated with an increased risk of reported pancreatitis in France [9]. In Chen’s meta-analysis, an increased risk of acute pancreatitis was related to DDP-4i drugs in patients with type 2 diabetes [28]. According to a population-based matched case-control study, the risk factors of DPP-4i associated pancreatitis were hypertriglyceridemia, alcohol use, gallstones, tobacco abuse, obesity, biliary and pancreatic cancer, cystic fibrosis, and neoplasms [29]. On the other hand, patients with T2DM generally have a higher risk of acute pancreatitis and pancreatic cancer, mainly if they suffer from chronic pancreatitis, which may explain the significant signals detected in DPP-4is. Besides, the risk of pancreatic cancer is much more challenging to assess, given that it is hard to perform a much longer follow-up in a clinical trial. Also, the ROR value is overestimated by the “notoriety bias” (increased reporting of adverse drug events following safety alerts by regulatory authorities) as a result of the FDA alerts [30]. Therefore, it is difficult to assess the possible effect of DPP-4i. This was also the conclusion of a recent assessment by the US FDA and the European Medicines Agency, who further commented that while data provide reassurance, pancreatitis will continue to be considered a risk associated with incretin-based therapies until more data are available [26]. Given the potential risk of pancreatitis in diabetic patients using DPP-4 inhibitors, pre-existing risk factors for pancreatitis should be taken into account when prescribing this type of medication.

DPP-4 enzyme has been implicated to have a direct effect in T lymphocyte regulation [18], and DPP-4i suppress mitogen-stimulated T-cell responses [31], which leads to an increased incidence of infections (e.g., upper respiratory tract infection, nasopharyngitis, and urinary tract infection) [20, 32]. A case/non-case study in the World Health Organization VigiBase indicates an increased reporting of the three infections [33]. Similarly, our research suggests increased reporting of these infections and pneumonia for users of DPP-4 inhibitors (as a class) compared with users of other non-insulin antidiabetic drugs. Meta-analyses suggested an increased risk for all-cause infections [34, 35]. However, these results are questionable. There are also systematic reviews and meta-analyses indicated that no notable between-group differences in incidence rates were observed for the three infection-related adverse events [36, 37].

Consistent with previous disproportionality studies [38], SDR was observed in arthralgia and myalgia. Besides, “pain in extremity” also showed significant disproportionality in our study. The underlying mechanism for these ADRs could be explained by the wide distribution of DPP-4 in striated muscle. DPP-4i increases the levels of P substance (thus decreasing the pain threshold) and slightly increases endomorphin-2 levels [39]. For the fracture, the observations that patients with T2DM are at an increased risk of bone fractures have led to increased vigilance regarding these events and the interaction with various therapies, including the DPP-4i [40]. However, in the SAVOR-TIMI 53 trial, the risk was similar in each treatment group [41], and a meta-analysis indicated that DPP-4i might reduce the risk of bone fractures [42]. SDR was not detected for fracture in our study. Therefore, the significant association was observed in the disproportionality analyses for musculoskeletal disorders, despite not being severe.

The effect of antidiabetic agents on cardiac outcomes has been a matter of uncertainty for the past four decades [43]. Thus, in 2008 the FDA issued a guidance document calling for the cardiovascular safety assessment of all new glucose-lowering therapies [44]. Early pharmacovigilance research found an association for an increased risk of reported cardiac disorders during DPP-4i exposure [45]. In the SAVOR-TIMI 53 trials, saxagliptin increased hospitalization rate for heart failure (3.5% versus 2.8%; hazard ratio, 1.27; 95% confidence interval, 1.07–1.51) in patients with T2DM [46]. Subsequently, a meta-analysis and a large cohort study provided additional evidence that saxagliptin was not associated with hospitalization for heart failure [47, 48]. Recent pharmacovigilance research indicated the association between cardiac complications and DPP-4is was biased by stimulated reporting [8], and the risk of cardiac disorders related to DPP-4is was non-significant after adjusted. Based on the molecular mechanism, DPP-4 inhibitors play a critical role in vascular and vessel protection through preventing cleavage of stem cell chemoattractant cytokine and enhancing the homing of endothelial progenitor cells [49]. Our study did not detect the signals for cardiac disorders (cardiac failure, cardiac failure congestive, and myocardial infarction), which agrees with the clinical trial [46]. However, continued investigation is needed to define the results.

Postmarketing events of hypersensitivity reactions, including anaphylaxis, rash, and angioedema, are reported in the prescribing information of most DPP-4is [50]. And consistent with postmarket data, we found SDRs for angioedema, pruritus, rash, rash generalized, and urticarial. Previous pharmacovigilance study in the VigiBase found disproportionality signal for an increased risk of bullous pemphigoid during DPP-4 inhibitor exposure, similar to our study conducted in FAERS. The author speculated that all DPP-4 inhibitors are selective for the DPP-4 enzyme, and the affinity for the DPP-4 enzyme may contribute to the risk of bullous pemphigoid. The affinity data of a DPP-4i drug for the enzymes explain to some extent different levels of bullous pemphigoid reporting in our study (quantified as ROR) [7].

The FAERS database is considered a valuable tool; however, some limitations inherent to spontaneous reporting have been noted. First, the database is increasingly incomplete (e.g., missing patient demographic information), variable reporting rates over time, duplicate reports, an unverified source of submitted data, inability, and missing information [51]. To overcome the limitation, reports were cleaned before analysis. Second, there are inherent limitations to establishing causal relationships, including the evaluation of events with high background rates, long latency periods, notoriety bias, or a possible contribution by coexisting medical conditions or comedications [46]. Also, reports from FAERS are not medically confirmed, which may introduce reporter bias [52]. Therefore data mining does not provide sufficient evidence on causality and merely suggests the necessity for practitioner vigilance. However, we attempted to alleviate this bias by limiting the analysis to a population of patients with diabetes (using non-insulin antidiabetic agents as proxy), which presumably share at least a set of common risk factors. Despite some limitations inherent to spontaneous reporting, the FAERS database is a rich resource. Data mining is one of the critical tools for routine assessment and management of risks associated with marketed pharmaceutical products.

## Conclusions

The safety profiles of sitagliptin, saxagliptin, linagliptin, and vildagliptin were reviewed using AERs submitted to the FAERS. Among non-insulin antidiabetics, DPP-4is are associated with higher AEs reporting of the gastrointestinal tract, pancreatitis, malignancies, infection, musculoskeletal system, general disorders, hypersensitivity and skin, corroborating clinical trial evidence. Furthermore, a signal is not detected for cardiopathy and fracture. Our findings need further validation and should be interpreted with caution, given the limitations of the pharmacovigilance. However, for physicians, these possible associations should be aware, and the patient’s comorbidities and history and potential adverse effects of the medicine must be taken into consideration. Future research needs to focus on safety concerns, especially the development of cancer and pancreatitis. Finally, our study provides a better understanding of the safety profiles of DPP-4i in a pharmacovigilance way.

## Supplementary information

**Additional file 1: Supplementary Table 1.** fourfold table for measure of disproportionality.

## Data Availability

The datasets generated and analyzed during the current study are available from the corresponding author on reasonable request.
