# Supplementary material for: Adverse event profiles of dipeptidyl peptidase-4 inhibitors: data mining of the public version of the FDA adverse event reporting system
Source: BMC Pharmacol Toxicol. 2020 Sep 16;21:68. doi: 10.1186/s40360-020-00447-w (PMC7493367; doi:10.1186/s40360-020-00447-w)
Supplement: Supplementary file 1 — Additional file 1: Supplementary Table 1. fourfold table for measure of disproportionality. [file 40360_2020_447_MOESM1_ESM.docx]

Supplementary Material 1

Table 1 fourfold table for measure of disproportionality

|  | Adverse event of interest | All other adverse events | Total |
| --- | --- | --- | --- |
| Dipeptidyl peptidase-4 inhibitors | a | b | a+b |
| Comparison group | c | d | c+d |
| Total | a+c | b+d | a+b+c+d |

Comparison groups: non-insulin antidiabetic drugs

ROR ＝(a×d)/(b×c) 95%CI＝e^ln(ROR)±1.96√(1^*^/a+^*^1^*^/b+^*^1^*^/c+^*^1^*^/d)^*
